# Genetic loci for resistance to podocyte injury caused by the tensin2 gene deficiency in mice

**DOI:** 10.1186/s12863-018-0611-1

**Published:** 2018-04-10

**Authors:** Yuki Takahashi, Hayato Sasaki, Shiori Okawara, Nobuya Sasaki

**Affiliations:** Laboratory of Laboratory Animal Science and Medicine, School of Veterinary Medicine, Kitasato University, 35-1, Higashi-23, Towada, Aomori, 034-8628 Japan

**Keywords:** Tensin2, Albuminuria, Podocyte, Linkage analysis

## Abstract

**Background:**

Tensin2 is a focal adhesion-localized multidomain protein expressed in various tissues, and its dysfunction leads to alterations in podocytes. However, these podocyte-related manifestations are dependent on murine strain. Tensin2 dysfunction results in susceptible strains developing podocyte foot process effacement and massive albuminuria, whereas podocytes in resistant strains remain almost intact. In our previous studies, quantitative trait loci analysis and congenic analysis using resistant C57BL/6J and susceptible ICGN mice identified a modifier locus associated with podocyte injury caused by tensin2 dysfunction on chromosome 2. However, the effect of this modifier locus on chromosome 2 is insufficient to explain the resistance of C57BL/6J mice to tensin2 dysfunction, indicating the existence of other modifier genes.

**Results:**

Whereas previous studies focused on the severity of chronic kidney disease, the present study focused on podocyte injury. We performed a genome-wide linkage analysis of backcrosses between two tensin2-deficient mouse strains, B6.ICGN-*Tns2*^*nph*^ and FVB.ICGN-*Tns2*^*nph*^, and detected a novel major modifier locus on chromosome 10. The combined effect of the C57BL/6J alleles of the two loci on chromosomes 2 and 10 reduced the urinary albumin excretion caused by tensin2 dysfunction to a level comparable to that of C57BL/6J mice.

**Conclusions:**

These data indicate that the resistance to podocyte injury caused by tensin2 dysfunction is mainly produced by the effects of the modifier genes on the two loci. The identification of these modifier genes is expected to help elucidate the mechanism underlying podocyte injury.

**Electronic supplementary material:**

The online version of this article (10.1186/s12863-018-0611-1) contains supplementary material, which is available to authorized users.

## Background

Tensin2 (Tns2), a member of the tensin family that includes Tns1, Tns3 and Tns4, is a focal adhesion-localized multidomain protein that possesses protein tyrosine phosphatase (PTP) and Src homology 2 (SH2)-phosphotyrosine binding (PTB) domains [1]. *Tns2* mRNA is detected in the brain, heart, kidney, skeletal muscle, liver, lung, colon, small intestine and other tissues [2–6]. Stable expression of Tns2 in HEK293 cells alters cell morphology and reduces actin filaments. Overexpression of Tns2 in HEK293 cells reduces cell proliferation in a functional PTP domain-dependent manner and inhibits cell migration [7]. In contrast, stable expression of Tns2 protein with its C terminus fused to green fluorescent protein in HEK293 cells promotes cell migration [2]. In a podocyte cell line, it was shown that *Tns2* knockdown enhances actin stress fiber formation and cell migration [8]. Taken together, these studies suggest that Tns2 function is involved in the reorganization of the actin cytoskeleton.

However, in vivo, Tns2 dysfunction only leads to apparent abnormalities in the kidney glomerulus [4, 8]. Newborn ICGN mice, carrying a Tns2-null mutation (hereafter *nph*), showed normal glomerular development, whereas podocyte foot process effacement and abnormal thickening of the glomerular basement membrane (GBM) were observed by 14 days after birth [9]. GBM thickening, which begins during glomerular maturation in ICGN mice, is considered to be a result of the abnormal extracellular matrix accumulation caused by podocytes but not by endothelial cells [9]. As observed in chronic kidney disease, ICGN mice then develop glomerular injury, including mesangial expansion, and massive albuminuria followed by tubulointerstitial injury with age [10, 11].

The podocyte-related manifestations of Tns2 dysfunction also depend on murine strain. For example, in response to *nph* mutation, albuminuria or podocyte foot process effacement was observed in FVB/NJ (FVB) and DBA/2J, but not in C57BL/6J (B6) and 129^*+Ter*^/SvJcl mice [12–16]. This disparity among strains indicates the presence of modifier genes. A comparison of the differences in the genetic backgrounds between the Tns2-deficiency resistant and susceptible strains can help in identifying these modifier genes, which may also help in elucidating the mechanism underlying Tns2 function in podocytes. Indeed, we previously performed quantitative trait loci (QTL) analysis and congenic analysis using resistant B6 and susceptible ICGN mice, and identified a modifier locus associated with podocyte injury caused by the *nph* mutation, *Tpir*, on chromosome 2 [17, 18]. ICGN.B6 congenic strains carrying the B6 allele of *Tpir* showed alleviated podocyte foot process effacement, GBM thickening and albuminuria, although the degree of improvement was inferior to that in the B6 genetic background itself, which shows almost no podocyte alteration [18]. This result indicates the existence of other modifier genes. The following points may explain why our previous QTL analysis failed to find the other significant loci: (1) our previous analysis focused on the severity of chronic kidney disease, and evaluated the traits related to the late-phase of chronic kidney disease in 16-week-old mice, such as tubulointerstitial injury, renal anemia and renal failure, but not albuminuria; (2) the quantitative traits, except for the histological scores, varied greatly in the ICGN mice themselves; and (3) the glomerular injury score focused on mesangial matrix accumulation, which is primarily caused by abnormal extracellular matrix metabolism in mesangial cells [19]. A further problem is that complete genetic information on the ICGN mouse genome remains unavailable.

In this study, we focused on podocyte injury and undertook a genome-wide linkage analysis of backcrosses between two Tns2-deficient mouse strains, B6.ICGN-*Tns2*^*nph*^ (B6-*Tns2*^*nph*^) and FVB.ICGN-*Tns2*^*nph*^ (FVB-*Tns2*^*nph*^) to identify modifier loci that may have been missed in previous studies.

## Methods

### Animals

**Fig. 1:**
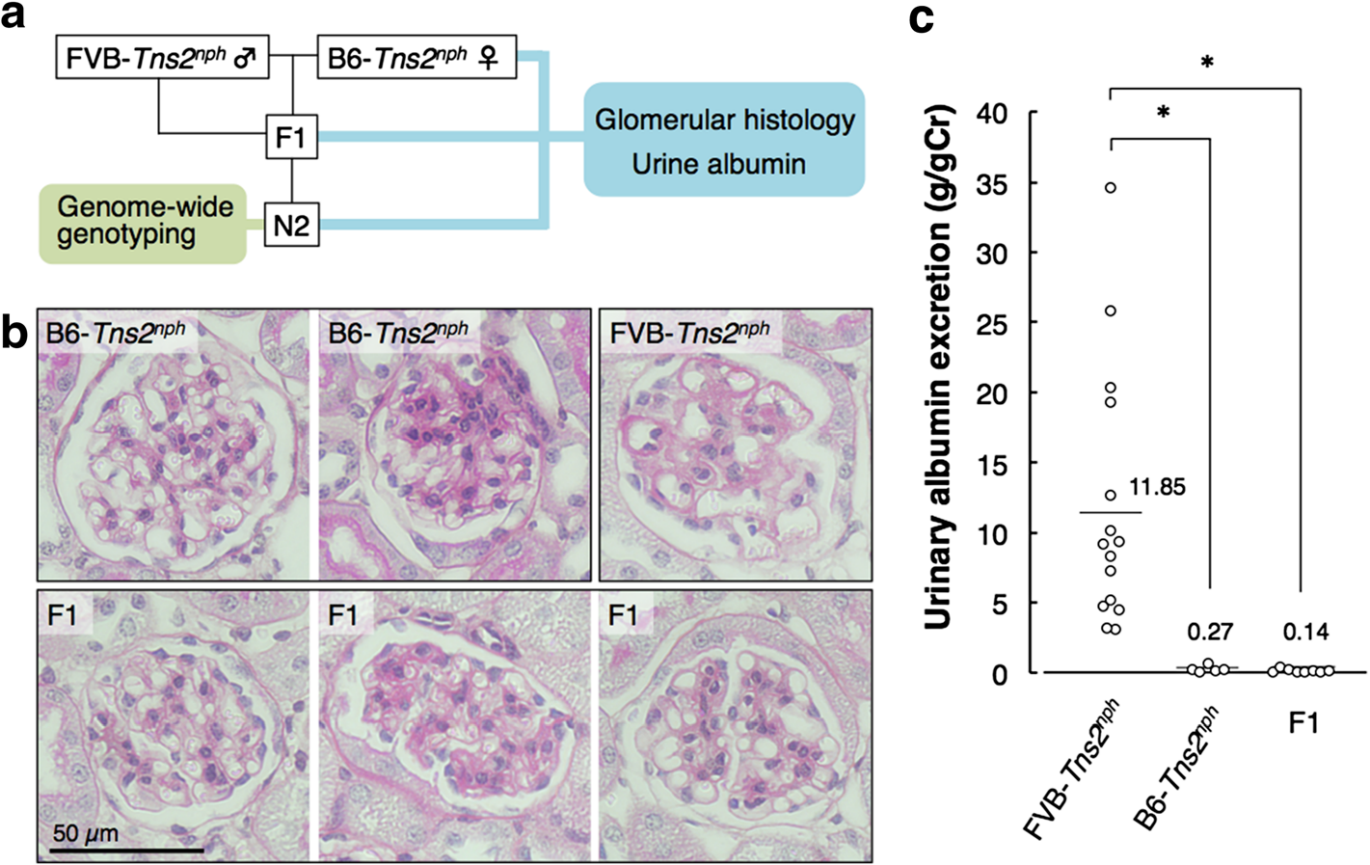
Glomerular histology and urinary albumin excretion in Tns2-deficient B6, FVB and F1 mice. (**a**) Schema of the study. **b** Representative glomerular structures in PAS staining. In B6-*Tns2*^*nph*^ mice, most glomeruli were normal (left panel), while some glomeruli showed a slight increase in mesangial matrix (right panel). In FVB-*Tns2*^*nph*^ mice, many glomeruli showed marked expansion of the mesangial matrix, marked thickening of the GBM and abnormal dilation of the capillary lumen. In F1 mice, there were normal glomeruli (left panel), glomeruli with mesangial expansion (middle panel) and glomeruli with GBM thickening (right panel). **c** Urinary albumin excretion in FVB-*Tns2*^*nph*^ (*n* = 15), B6-*Tns2*^*nph*^ (*n* = 5) and F1 (*n* = 8) mice. Values represent the mean. Asterisk, *P* < 0.01

B6-*Tns2*^*nph*^ and FVB-*Tns2*^*nph*^ congenic mice were generated as described previously [12, 15]. B6 x FVB F_1_ hybrid *Tns2*^*nph*^ (F1) mice were obtained by mating female B6-*Tns2*^*nph*^ mice to male FVB-*Tns2*^*nph*^ mice. N2 backcross *Tns2*^*nph*^ progenies were then obtained by mating female F1 mice to male FVB-*Tns2*^*nph*^ mice (Fig. 1a). The SPF room was air-conditioned at 22 ± 4 °C, maintained at 40–60% relative humidity, and mice were maintained under a 12 h light-dark cycle. A standard laboratory diet, CE-2 (CLEA Japan, Tokyo, Japan), and tap water were provided ad libitum.

### Genotyping

Genome-wide genotyping of the *Tns2*^*nph*^ N2 mice was performed using 99 polymorphic markers (Additional file 1: Table S1). The *Tyr* locus was genotyped by coat color, and the other loci were genotyped using polymerase chain reaction (PCR)-based method. Genomic deoxyribonucleic acid was extracted from ear tissue obtained during ear punching for identification. PCR was performed with the following thermal cycling: initial denaturation at 95 °C for 3 min; 10 cycles of 95 °C for 10 s, 65 °C for 30 s (reduced by − 1 °C/cycle), and 72 °C for 10 s; and 30 cycles of 95 °C for 10 s, 55 °C for 30 s, 72 °C for 10 s. Newly designed PCR primers are listed in the Additional file 1: Table S2.

### Measurement of urinary albumin excretion

Urine samples were collected from 8-week-old mice by gentle manual compression of the abdomen. To measure albumin, urine samples were diluted with sample buffer containing 2% sodium dodecyl sulfate (SDS), 5% ß-mercaptoethanol, 10% glycerol, 60 mM Tris-HCL (pH 6.8) and bromophenol blue, and heated at 95 °C for 5 min. Samples containing 1 μL of urine were applied to 10% SDS-polyacrylamide gel electrophoresis. As a positive control, 5 μg of bovine serum albumin was loaded simultaneously. The gel was fixed and stained with Coomassie brilliant blue (CBB; Nacalai Tesque, Kyoto, Japan) according to manufacturer’s instructions, and scanned using a standard commercial scanner. CBB-stained urinary albumin was quantified by the ImageJ gel analysis program (http://imagej.net/). Urinary creatinine was measured using a creatinine colorimetric assay kit (Cayman chemical, Michigan, USA) according to manufacturer’s instructions. The urinary albumin excretion was normalized against the urinary creatinine. Urine collection was performed twice over a three-day period, and the measured urinary albumin excretion was averaged for each mouse.

### Histology

Kidneys from 8-week-old mice were fixed with 4% paraformaldehyde (PFA) at 4 °C overnight. The PFA-fixed paraffin sections (2 μm thick) were subjected to normal histological processes and stained with periodic acid-Schiff (PAS) solution.

### Marker-trait association test

N2 mice were divided into two groups, affected and not affected, according to their urinary albumin excretion. With reference to the distribution of urinary albumin excretion observed in FVB-*Tns2*^*nph*^ and B6-*Tns2*^*nph*^ mice, we defined the N2 mice with urinary albumin excretion larger than the minimum value in FVB-*Tns2*^*nph*^ mice as the FVB type (affected with albuminuria) and the N2 mice with urinary albumin excretion less than the maximum value in B6-*Tns2*^*nph*^ mice as the B6 type (not affected with albuminuria). Another way of division was to define the upper 30% or 40% or 50% of N2 mice as the FVB type and the lower 30% or 40% or 50% of N2 mice as the B6 type. A chi-square test was applied to detect marker-trait associations.

### Statistics

Data are expressed as means ± standard deviation. Student’s *t*-test and Dunnett’s multiple comparison test were used for comparisons of two or more than two independent groups, respectively. A *p* value < 0.05 was considered statistically significant. Dunnett’s multiple comparison test was carried out using GraphPad Prism 5 software (MDF, Tokyo, Japan).

## Results

### Phenotype of Tns2-deficient B6, FVB and (B6 x FVB) F_1_ mice

Consistent with previous reports [12–15], FVB-*Tns2*^*nph*^ mice showed albuminuria (urinary albumin excretion, 11.8 ± 9.2 g/gCr) and severe glomerular injuries, such as marked expansion of the mesangial matrix, marked thickening of the GBM and abnormal dilation of capillary lumen, whereas B6-*Tns2*^*nph*^ mice did not show either albuminuria (urinary albumin excretion, 0.26 ± 0.25 g/gCr) or glomerular injury, apart from a slight increase in the mesangial matrix in some glomeruli (Fig. 1b, c). To assess the mode of inheritance of traits for resistance to Tns2 dysfunction, F1 mice were subjected to similar analyses at 8 weeks. The structures of some glomeruli in the F1 mice were nearly normal (Fig. 1b); however, unlike that in B6-*Tns2*^*nph*^ mice, a focal thickening of the GBM was also observed in F1 mice (Fig. 1b), suggesting that F1 mice exhibited an intermediate phenotype for glomerular injury caused by Tns2 dysfunction. On the other hand, the urinary albumin excretion of F1 mice (0.14 ± 0.13 g/gCr) was approximately equal to that of B6-*Tns2*^*nph*^ mice (Fig. 1c), suggesting that resistance to albuminuria caused by Tns2 dysfunction is inherited as a dominant trait in F1 mice. Although the glomerular injuries may afford a sensitive reflection of the severity of renal disease caused by Tns2 dysfunction, we considered that urinary albumin excretion is a better indicator of resistance to Tns2 dysfunction than is glomerular injury, which is complicated and difficult to quantify.

### Genome-wide linkage analysis in N2 backcross mice

**Fig. 2:**
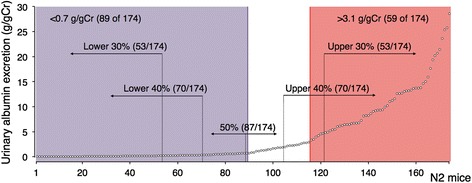
Distribution of urinary albumin excretion in N2 mice. Vertical lines illustrated in the figure represent the borders of the phenotype definitions for marker-trait association test. *n* = 174

**Fig. 3:**
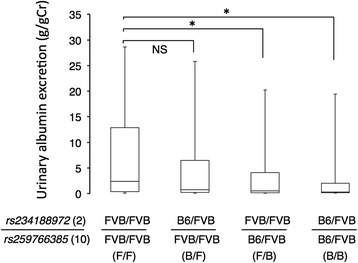
Effects of modifier loci on urinary albumin excretion in N2 mice. Urinary albumin excretion of N2 mice was divided by the four combined genotypes of *rs234188972* on chromosome 2 and *rs259766385* on chromosome 10. The numbers of groups were 47, 37, 47 and 43 for F/F, B/F, F/B and B/B, respectively. Bars represent the maximum and minimum values. Values represent the median. Asterisk, *P* < 0.05. NS, not significant

As F1 mice as well as B6-*Tns2*^*nph*^ mice exhibited a suppressive phenotype, we backcrossed the F1 mouse onto the FVB-*Tns2*^*nph*^ mouse to identify the modifier loci related to resistance to Tns2 dysfunction. We then performed genome-wide genotyping of the resultant N2 mice (*n* = 174) and subsequent phenotyping for albuminuria. N2 mice showed a continuous spectrum of values for urinary albumin excretion and did not simply segregate into the B6- and FVB-*Tns2*^*nph*^ types according to whether or not they were affected with albuminuria (Fig. 2). Therefore, with reference to the distribution of urinary albumin excretion observed in FVB-*Tns2*^*nph*^ (3.12–34.6 g/gCr) and B6-*Tns2*^*nph*^ mice (0.052–0.69 g/gCr), we defined the N2 mice with urinary albumin excretion larger than 3.1 g/gCr (59 of 174) as the FVB type (affected) and the N2 mice with urinary albumin excretion less than 0.7 g/gCr (89 of 174) as the B6 type (not affected). In the N2 mice as defined above, marker-trait associations were detected on chromosome 10. The genetic markers *rs259766385* and *rs239646663* on chromosome 10 showed a statistical significant relationship with albuminuria (Table 1). In addition, the analyses using different phenotype definitions (e.g., the upper 40% and lower 40% of N2 mice) found suggestive signals on chromosome 2 (Table 1). Furthermore, the combined *rs259766385* genotypes and the genetic markers on chromosome 2 provided a more significant *p* value in comparison with the chi-square test for each marker (Table 2). Table 2 shows a comparison between the two combined genotypes; one in which both the markers are homogeneic, the other in which at least one of the markers is heterogeneic. In Fig. 3, the variations in urinary albumin excretion were compared among the four combined genotypes for *rs234188972* on chromosome 2 and *rs259766385* on chromosome 10. Urinary albumin excretion in both groups with allelic heterogeneity at *rs259766385* (F/B and B/B groups) was significantly lower than that in the group carrying FVB/FVB genotypes at the two markers (F/F group) (Fig. 3). The median value for urinary albumin excretion in the group carrying B6/FVB and FVB/FVB genotypes at *rs234188972* and *rs259766385*, respectively (B/F group, 0.71 g/gCr), was approximately equal to those of the F/B and B/B groups (0.52 and 0.32 g/gCr, respectively) and appeared lower than that of the F/F group (2.4 g/gCr), although no significant difference was detected between the B/F and F/F groups (Fig. 3).

**Table 1:** Relationship between albuminuria and genotype for genetic markers on chromosomes 2 and 10

Chromosome	Marker	Position (cM)	χ^2^ (P)				Student-T (P)^c^			
			Min/Max^a^	50%^b^	40%^b^	30%^b^	Min/Max	50%	40%	30%
2	*D2Mit398*	61.76	0.19	0.062	0.052	0.20	0.48	0.47	0.40	0.31
2	*rs214164633*	65.66	0.16	0.080	0.075	0.20	0.26	0.25	0.24	0.18
2	*rs234188972*	70.98	0.18	0.067	0.062	0.24	0.14	0.16	0.14	0.10
2	*rs247793542*	75.41	0.64	0.36	0.30	0.84	0.25	0.23	0.26	0.24
2	*rs228447345*	81.91	0.65	0.28	0.44	1	0.42	0.45	0.49	0.49
10	*D10Mit298*	2.78	0.58	0.42	0.60	0.48	0.43	0.47	0.36	0.29
10	*D10Mit3*	16.53	0.010	0.053	0.086	0.04	0.0099	0.0084	0.012	0.013
10	*D10Mit197*	35	0.049	0.059	0.059	0.10	0.0078	0.011	0.012	0.014
10	*rs259766385*	40.66	0.0022	0.0039	0.0068	0.0065	0.00038	0.00098	0.00074	0.00061
10	*rs239646663*	43.64	0.0043	0.0099	0.0040	0.019	0.00053	0.00076	0.0010	0.0015
10	*D10Mit233*	61.58	0.054	0.092	0.042	0.079	0.14	0.17	0.20	0.15

^a^The N2 mice with urinary albumin excretion larger than 3.1 g/gCr, the minimum value in FVB-*Tns2*^*nph*^ mice, were defined as the FVB type (affected) and the N2 mice with urinary albumin excretion less than 0.7 g/gCr, the maximum value in B6- *Tns2*^*nph*^ mice, were defined as the B6 type (not affected)

^b^The upper X% and lower X% of N2 mice in urinary albumin excretion were defined as the FVB type (affected) and the B6 type (not affected), respectively

^c^Urinary albumin excretion in N2 mice was compared between those with B6/FVB and FVB/FVB genotypes at the genetic markers

**Table 2:** Relationship between albuminuria and combined genotypes of chromosomes 2 and 10 (*rs259766385*)

Chromosome	Marker	Position (cM)	χ^2^ (P)				Student-T (P)^c^			
			Min/Max^a^	50%^b^	40%^b^	30%^b^	Min/Max	50%	40%	30%
2	*D2Mit398*	61.76	0.003	0.0019	0.0049	0.014	0.055	0.078	0.075	0.027
2	*rs214164633*	65.66	0.0013	0.0012	0.0027	0.046	0.0016	0.0045	0.0028	0.0010
2	*rs234188972*	70.98	0.0013	0.00090	0.0032	0.0097	0.00021	0.0015	0.00083	0.00025
2	*rs247793542*	75.41	0.0063	0.0054	0.012	0.024	0.0020	0.0070	0.0042	0.0013
2	*rs228447345*	81.91	0.0039	0.0021	0.012	0.10	0.0089	0.028	0.025	0.015

^a^The N2 mice with urinary albumin excretion larger than 3.1 g/gCr, the minimum value in FVB-*Tns2*^*nph*^ mice, were defined as the FVB type (affected) and the N2 mice with urinary albumin excretion less than 0.7 g/gCr, the maximum value in B6- *Tns2*^*nph*^ mice, were defined as the B6 type (not affected)

^b^The upper X% and lower X% of N2 mice in urinary albumin excretion were defined as the FVB type (affected) and the B6 type (not affected), respectively

^c^Urinary albumin excretion in the homogeneic genotype, in which both markers were FVB/FVB genotype, were compared with those in the heterogeneic genotype, in which at least one of the markers was B6/FVB genotype

## Discussion

In this study, to identify the modifier loci in podocyte alterations caused by Tns2 dysfunction, we assessed the relationship between albuminuria phenotype and genotype in backcrosses between resistant B6-*Tns2*^*nph*^ and susceptible FVB-*Tns2*^*nph*^ mice. Initially, we demonstrated that F1 mice exhibited an intermediate phenotype for glomerular injury between resistant B6 and susceptible FVB types, while little of no urinary albumin was detected in both the F1 mice and B6-*Tns2*^*nph*^ mice. The results indicated that heterozygous alleles at the modifier loci show an intermediate phenotype for podocyte injury, leading to an increase in albumin leakage through the glomerular filtration barrier. However, F1 mice showed a resistant B6-type phenotype for albuminuria, probably due to the proximal tubular reabsorption of albumin [20].

Subsequently, F1 mice were backcrossed to FVB-*Tns2*^*nph*^ mice. The N2 backcrosses showed a wide spectrum of urinary albumin excretion ranging from B6 type to FVB type, and including intermediate values. These values indicated that some N2 mice exhibited podocyte and glomerular injuries that were more severe than those of F1 mice but milder than those of FVB-*Tns2*^*nph*^ mice. These injuries are expected to lead to greater albumin leakage, which cannot be reabsorbed by the proximal tubules, resulting in a continuous spectrum of values for urinary albumin excretion in N2 mice. These results also indicate that there are multiple modifier loci for podocyte injury caused by Tns2 dysfunction. However, a QTL analysis of urinary albumin excretion failed to identify any modifier loci (data not shown). We assumed that this is because that the intended modifier effects are hindered due to a wide spectrum of values for urinary albumin excretion in affected mice themselves (see Fig. 1c). Therefore, we simply segregated N2 mice into two groups, affected and not affected, and examined the specific relationship between genotype and phenotype using chi-square test. Then, a marker-trait association test suggested the existence of two loci, a significant locus on chromosome 10 and a suggestive locus on chromosome 2, whose peak marker *rs234188972* is included in *Tpir* (57.65–78.72 cM). B6 homozygous alleles of *Tpir* exerted a protective effect against podocyte alterations and reduced urinary albumin excretion in affected mice to approximately 50% [18]. In this study, B6 heterozygous alleles of the suggestive locus on chromosome 2 were observed to partially alleviate podocyte injury, based on the results shown in Fig. 3. We, therefore, speculated that a causative gene at the suggestive locus is identical to *Tpir*, and we designated this suggestive locus as *Tpir1*.

As the protective effect of the B6 allele of *Tpir* was insufficient in comparison with that of the B6 genetic background itself, our previous study predicted the existence of an as yet unidentified major modifier locus [18], which actually corresponds to the significant locus identified on chromosome 10. B6 heterozygous alleles of the significant locus markedly reduced urinary albumin excretion in affected mice. Furthermore, B6 heterozygous alleles of both *Tpir1* and the significant locus exerted a protective effect on albuminuria at levels close to that of the B6 genetic background. We, therefore, designated the significant locus as *Tpir2*. No genes located around *Tpir1* and *Tpir2* are known to be involved in nephropathy.

**Fig. 4:**
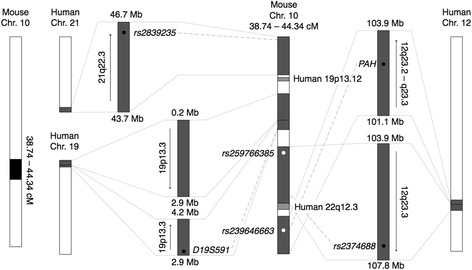
Schematic representation of the genomic region of mouse chromosome 10 at 38.74–44.34 cM and the homologous regions in human. The genomic regions of mouse chromosome 10 at 38.74–44.34 cM are syntenic to human 12q23.2 – q23.3, 19p13.3, 19p13.12, 21q22.3 and 22q13.3. White dots represent mouse genetic markers used in this study: *rs259766385* and *rs239646663* are the peak markers of *Tpir2*. Black dots represent human genetic markers: *rs2374688* and *rs2839235* are the single nucleotide polymorphisms associated with urinary albumin excretion and glomerular filtration rate in human, respectively [21]; and *PAH* and *D19S591* (also known as *GATA44F10*) are the genetic markers associated with urinary albumin excretion in patients with essential hypertension [22]. Chr., chromosome

The genomic region of chromosome 10 at 38.74–44.34 cM including *rs259766385*, the peak marker of *Tpir2*, consists of multiple syntenic regions on human chromosomes 12, 19, 21 and 22. It is notable that the syntenic regions on human 12q23.2 – q23.3 and 19p13.3, and 21q22.3 include the genetic markers associated with urinary albumin excretion (*PAH*, *rs2374688*, *D19S591*) and glomerular filtration rate (*rs2839235*) (Fig. 4) [21, 22]. It is interesting that the multiple genetic markers associated with urinary albumin excretion or glomerular filtration rate on the different human chromosomes are located on a single syntenic 5.6-cM interval (38.74–44.34 cM) of mouse chromosome 10, albeit including a gene-rich region (39.72-cM) that encompasses 233 genes. This suggests that there might be multiple modifier genes on *Tpir2*. The *Tpir2* genes located on syntenic human 12q23.2 – q23.3, 19p13.3 and 21q22.3 are shown in the Additional file 1: Table S3.

## Conclusions

Our present analysis identified two genetic regions associated with podocyte injury induced by Tns2 dysfunction, *Tpir1* and *Tpir2*, which are on chromosomes 2 and 10, respectively. *Tpir1*, which is considered to be identical to *Tpir*, the genetic region identified by our previous study, is insufficient to explain the resistance of the B6 genetic background to Tns2 dysfunction, while *Tpir2* is a major modifier locus for this resistance. The combined protective effect of the B6 alleles of *Tpir1* and *Tpir2* was comparable to that of the B6 genetic background itself. The identification of these modifier genes is expected to help elucidate the mechanism underlying podocyte injury.

## Additional file

Additional file 1: **Table S1.** Genomic positions of polymorphic genetic markers between B6 and FVB strains. **Table S2.** Sequences of the PCR primers used to identify polymorphic indel mutations between B6 and FVB strains. **Table S3.** Genes located on syntenic human 12q23.2 – q23.3, 19p13.3 and 21q22.3. (XLSX 28 kb)

## Abbreviations

CBB: Coomassie brilliant blue
CKD: Chronic kidney disease
cM: Centimorgan
GBM: Glomerular basement membrane
gCr: Gram creatinine
PAS: Periodic acid-Schiff
PCR: Polymerase chain reaction
PFA: Paraformaldehyde
PTB: Phosphotyrosine binding
PTP: Protein tyrosine phosphatase
QTL: Quantitative trait loci
Rho-GAP: Rho GTPase-activating protein
SDS: Sodium dodecyl sulfate
SH2: Src homology 2
